# The impact of COVID-19 post-infection on the cognition of adults from Peru

**DOI:** 10.3389/fpsyg.2024.1325237

**Published:** 2024-06-25

**Authors:** Jonathan Zegarra-Valdivia, Harold Arana-Nombera, Leandro Perez-Fernandez, Reyna Alamo-Medina, Milagros del Rocío Casimiro, Diana Bustamante-Delgado, Maribel Matallana-Sanchez, Viviana Gallegos-Manayay, Elizabeth Álvarez-Bravo, Tihany Arteaga-Cancino, Eduardo Abanto-Saldaña, María del Rosario Oliva-Piscoya, María Celinda Cruz-Ordinola, Patricia Chavarry, Brenda Chino-Vilca, Carmen Paredes-Manrique, Carlos Chirinos, Nilton Custodio, Agustín Ibañez

**Affiliations:** ^1^Facultad de Ciencias de la Salud, Universidad Señor de Sipán, Chiclayo, Peru; ^2^Achucarro Basque Center for Neuroscience, Leioa, Biscay, Spain; ^3^Center of Cognitive and Computational Neuroscience-UCM, Madrid, Spain; ^4^Facultad de Humanidades, Universidad Tecnológica del Perú, Peru; ^5^Instituto Peruano de Neurociencias, Lima, Peru; ^6^Escuela Profesional de Medicina Humana, Universidad Privada San Juan Bautista, Lima, Peru; ^7^Global Brain Health Institute, University of California, San Francisco, San Francisco, CA, United States; ^8^Trinity College Dublin, Dublin, Ireland; ^9^Latin American Brain Health Institute (BrainLat), Universidad Adolfo Ibañez, Santiago, Chile

**Keywords:** COVID-19, cognitive performance, executive function, gender, adults

## Abstract

**Introduction:**

The COVID-19 pandemic, with over 83 million confirmed cases and 1.8 million deaths, has raised concerns about long-term cognitive issues, especially in populations facing disparities. Despite a few years since Peru’s first COVID-19 wave, the cognitive effects on adults remain unclear. This study is the first in Peru to explore COVID-19’s impact on general cognition and executive function.

**Methods:**

A retrospective cross-sectional study compared individuals with COVID-19 history to controls, assessing general cognition, verbal fluency, attention, and executive function. Among 240 assessed, 154 met the study inclusion criteria, with about 60% female and an average age of 38.89 ± 16.001 years. Groups included controls (*n* = 42), acute phase (AP, *n* = 74) (1–14 days of symptoms), and hyperinflammatory phase (HP, *n* = 38) (>14 days of symptoms).

**Results:**

Significant cognitive differences were observed. The HP group exhibited lower general cognitive performance (*p =* 0.02), working memory (*p =* 0.01), and executive function (planning; *p <* 0.001; flexibility; *p =* 0.03) than controls. Those with <14 days of illness (AP vs. HP) had deficits in general cognitive performance (*p =* 0.02), working memory (*p =* 0.02), and planning (*p <* 0.001), mainly during the hyperinflammatory phase, showing differences in working memory (*p =* 0.003) and planning (*p =* 0.01). Gender differences emerged, with males in the HP phase having poorer working memory (*p =* 0.003) and planning (*p =* 0.01).

**Discussion:**

This study underscores COVID-19’s negative impact on cognitive function, even in mild cases, with potential heightened effects in men during acute or hyperinflammatory phases. The findings provide Peru’s first evidence, highlighting the vulnerability of populations facing socioeconomic disparities.

## 1 Introduction

The COVID-19 conveyed 83 million confirmed cases and 1.8 million deaths (Wiersinga et al., 2020). Nowadays, more than 769 million cases are confirmed, with almost 7 million deaths by the World Health Organization (WHO) (WHO, 2023). The pandemic reached Latin America (LA) through different countries, and its impact has been pervasive, given the significant inequalities of the region (Ibanez et al., 2020; Ibanez and Kosik, 2020; Mena et al., 2021). By March 2020, it reached Peru (Cevik et al., 2020). The Peruvian government established a national state of emergency, including a mandatory nationwide quarantine, which caused a reduction in the mobility of people and closed non-essential productive activities (Hernández-Vásquez et al., 2022), impacting the perception susceptibility of the population about COVID-19, increasing fear and stigma, distrust the National Health administration’s response by the lack of adequate measures to deal with the emergency (Zegarra-Valdivia et al., 2022, 2023). However, the precarious health system, the informality of the labor market, and the increasing flow of migrants and vulnerable Indigenous communities in the Amazon (Vázquez-Rowe and Gandolfi, 2020) could be explained by the fact that nearly a million COVID-19-positive cases were detected with antibody tests (928016) by June 17, 2021, and 189,757 had died (MINSA M de S. Sala Situacional COVID-19, 2022).

After SARS-CoV-2 infection, the virus principally causes respiratory disease. However, neurological manifestations were also reported early in the pandemic, from worsening migraine symptoms (Reyes-Alvarez et al., 2023) to acute cerebrovascular events and other central and peripheral nervous system diseases (Ellul et al., 2020; Zegarra-Valdivia et al., 2020). Fatigue and cognitive dysfunction (brain fog, memory issues, attention disorder) were key neurological features present in roughly one-third of patients assessed 3 months after the onset of acute COVID-19 disease. Atypical pneumonia and strong autoimmune response left lingering effects that are still progressively clear (Yelin et al., 2020). The interaction between the human body and the virus on a respiratory and systemic level promotes fatal physical complications. However, the effects on the brain health of survivors are still pending (Kumar et al., 2021). The coronavirus is responsible for severe acute respiratory syndrome type 2 (SARS-CoV-2), which leads to neurological damage, such as delirium, strokes, encephalitis, and neuromuscular disorders (Zubair et al., 2020). It showed a similar effect to earlier coronaviruses (Zegarra-Valdivia et al., 2020).

Vascular endothelium damage, surrounding inflammation, and thrombosis have been confirmed (Solomon, 2021). The entry pathway was the olfactory mucosa, and the cellular approach mechanism was through the spike protein of SARS-CoV-2, which binds to the angiotensin-converting enzyme 2 (ACE2) receptor found in neurons and glia, resulting in mitochondrial alterations (Fotuhi et al., 2020; Wang et al., 2020). Autopsies showed inflammation-related changes with elevated levels of cytokines in the blood (Matschke et al., 2020). Patients often experience cognitive symptoms in isolation or combined with other neurological symptoms.

Moreover, neurological sequelae include headaches, and loss of smell and taste. In severe cases, up to a third of patients had neurological or psychiatric symptoms within 6 months of a COVID-19 diagnosis (Taquet et al., 2021). Cognitive and neurologic sequelae, including attention and executive deficits, are being studied (Woo et al., 2020; Davis et al., 2021). These results show a significant global disease burden, and the long-term prognosis for patients with coronavirus disease 2019 (COVID-19) after recovery stays unclear (Wang et al., 2023).

One explanation for the significant brain effects is that coronaviruses have an affinity for cerebrospinal fluid disrupting the blood-CSF barrier or are more related to cognitive post-acute sequelae (Apple et al., 2022). However, mechanisms of neurological infection were proposed (Zegarra-Valdivia et al., 2020) and are still under investigation (Wu et al., 2020). The theory of brain inflammation and clinical severity does not supply certainty for efficient diagnosis, which is why standardized criteria are necessary. Different studies in this field have varying inclusion criteria, timing, and methodologies, including control groups and longitudinal studies (Altuna et al., 2021).

Meanwhile, the mandatory lockdown during the pandemic also provoked an increase in the prevalence of Neuropsychiatric symptoms (NPS) (Premraj et al., 2022). A longitudinal study from Lima (Perú) reported new onset or worsening of NPS and cognitive decline in an MCI and AD patient’s cohort after the COVID-19 mobility restrictions (Custodio et al., 2021). While the same sample experienced stability or improvement in their NPS when the lockdown finished, the cognitive decline persisted over time (Custodio et al., 2023). The lockdown restrictions increased feelings of loneliness, decreased physical and mental activity, and reduced access to care directly or indirectly led to observed changes in cognitive function and NPS.

Another aspect to consider is the impact of the pandemic on the vulnerable population. In LA, particularly Peru, low education and illiteracy levels stay among the highest in the region. The distribution of the high and extremely high vulnerability index in rural provinces located or bordering the Andes Mountains increases the lower access to health services and other public services and unfavorable socioeconomic conditions in the populations in the mountainous regions (Zegarra Zamalloa et al., 2022). The high prevalence of lower educational achievement, sociodemographic disparities, and ethnic and cultural diversity in LA could explain the deficient performance of illiterate and educated individuals on neuropsychological tests (Chino et al., 2022; Santamaria-Garcia et al., 2023; Zegarra-valdivia et al., 2023) and might contribute to misdiagnosis in non-clinic context (Arafat et al., 2016; Rosselli et al., 2022; Lopera et al., 2023). The main aim was to determine the impact of COVID-19 history on general cognition and executive function.

## 2 Methods

### 2.1 Study design

A retrospective cross-sectional study was conducted with a cohort of participants with a COVID-19 history, and controls were thoroughly evaluated. We employed a non-probabilistic sampling method within the city of Chiclayo. The participant recruitment process was enhanced through targeted advertisements and by capitalizing on the assessments carried out on adults by our research team in various public institutions, including colleges and hospitals. This strategic approach enabled us to effectively reach and engage a diverse group of individuals for our study.

### 2.2 Participants

We use a non-probabilistic and convenient sampling (Hernández-Sampieri et al., 2010). Two hundred forty participants were evaluated in Chiclayo, one major city in northern Peru. We employ the inclusion criteria: (1) The participants reported no previous history of brain damage, neurologic or psychiatric treatment; (2) Confirmed diagnosis of COVID-19 infection history by PCR/antigen test; (3) Medical evaluation re-confirming the COVID-19 infection and presence of symptoms related in the clinic history reported; (4) voluntary and consent participation; (5) Complete cognitive evaluation; (6) No report of multiple COVID-19 infections; (7) Control subjects were not diagnosed with COVID-19 and tested negative by PCR or antigen test. The participants gave written informed consent to participate in the study. We also considered excluding criteria such as subjects with doubtful diagnoses, psychiatric symptoms, and without complete cognitive evaluation.

We excluded participants with (1) absence of PCR/antigen tests or doubtful diagnoses and (3) withdrawn by incomplete evaluation and cognitive assessment. Our cohort was divided into healthy controls not infected by COVID-19 and participants infected with COVID-19; these subjects were stratified according to the number of days reported with symptoms in the acute phase (AP), between 1 and 14 days, or hyperinflammatory phase (HP) more than 14 days (Datta et al., 2020). Lastly, data from 154 participants was used in this study.

### 2.3 Neuropsychological assessment

All participants were screened using standardized diagnostic instruments and received a neuropsychological assessment to explore their cognitive functioning by using the following tests:

#### 2.3.1 Rowland universal dementia assessment scale

The Rowland universal dementia assessment scale (RUDAS) is a simple cognitive assessment tool that can be administered in 10 min. It consists of 6 components that assess memory, body orientation, visuospatial praxis, motor praxis, judgment, and verbal fluency. A lower score on the RUDAS indicates poorer cognitive performance. This test is designed for illiterate, low, and middle-educated participants and has been transculturally adapted and validated in Peru, showing good validation standards (Custodio et al., 2019, 2020). The increased score shows better performance.

#### 2.3.2 INECO frontal screening

INECO frontal screening (IFS) is a neuropsychological tool that assesses frontal lobe functions. It consists of eight subtests grouped into three categories: response inhibition, set-shifting, and working memory (Torralva et al., 2009). The response inhibition and set-shifting subtests evaluate the individual’s ability to shift from one mental set to another and to inhibit inappropriate responses. The working memory subtests assess the individual’s ability to temporarily store information and manipulate it to perform complex cognitive tasks. The IFS has been validated in patients with brain dysfunction, neurodegeneration, and neuropsychiatric disorders (Baez et al., 2014; Custodio et al., 2016; Fernández-Fleites et al., 2021). It is a reliable and valid tool for finding deficits in frontal lobe functions. The increased score shows better performance.

#### 2.3.3 Backward digit span test

This cognitive assessment tool measures working memory and attention. Besides, it’s a good measure of auditory rehearsal, temporary storage capacity in working memory, and the ability to transform and manipulate numeric information (Goldstein et al., 2019). It can be administered in a few minutes while the examiner will read a series of numbers to the participant. The participant is then asked to repeat the numbers in the same order. The examiner will start by reading a series of two numbers and gradually increasing the number of digits in the series. The test is discontinued when the participant cannot correctly repeat a series of numbers. Besides, it displays good clinical validity in the Peruvian context (Zegarra-Valdivia and Chino-Vilca, 2018; Zegarra-Valdivia and Vilca, 2019). An increased score indicates better performance.

#### 2.3.4 Trail-making test parts A and B

Trail-making test (TMT) is a good measure of attention, processing speed, set-shifting reasoning, cognitive flexibility, problem-solving, divided attention, and reasoning (Gurd et al., 2010). TMT-A involves connecting 25 sequentially numbered circles on a sheet of paper. At the same time, TMT-B requires connecting numbers and letters where the sequence proceeds from the first number to the first letter alphabetically, followed by the second and second letters. The latter task is notably more challenging (Drane et al., 2002). The individual’s score is determined by the time to complete each test. This test has shown good validity and reliability with normative data for Latin America and Perú (Hernández-Sampieri et al., 2010), as good clinical validity (Chino et al., 2022; Lopera et al., 2023), even in low-educated populations (Margulis et al., 2018). An increased time in the execution writes down worse performance.

### 2.4 Procedure

Initially, the purpose of the evaluation was explained to all research participants, and their consent to participate was requested. Subsequently, all subjects completed the questionnaires and were administered the battery of neuropsychological tests, which trained evaluators conducted in one session lasting approximately 2 h. In these analyses, we only focus on cognitive changes. We used adapted and validated cognitive tools to determine cognitive changes in subjects previously infected with SARS-CoV-2 after 3–30 months of the initial COVID-19 infection diagnosis. We use standardized tests meticulously crafted to assess cognitive domains frequently explored in neuropsychological evaluations. These tests and their corresponding results are systematically reported and detailed within the domains section of our results. For general cognition, we use the RUDAS-P, a general assessment that analyzes different domains: memory, body orientation, visuospatial praxis, motor praxis, judgment, and verbal fluency.

On the other hand, the INECO Frontal Screening assesses different aspects of executive function, including response inhibition, set-shifting, and working memory. We supplement these with the Backward Digit Span Test (BDS) and the Trail-Making Test (TMT) parts A and B. From this perspective, we aim to cover important domains: memory, orientation, language, working memory, inhibition, time of execution, and executive control.

### 2.5 Ethical statement

All participants were informed about the aims of this study and gave written informed consent. This study followed ethics guidelines and was approved by the local ethics committee (N.° 0086-27092022-CIEI). All data was collected in an anonymous database, and no financial incentive was granted to the participants.

### 2.6 Statistical analysis

Our cohort was divided into controls and participants with previous history of COVID-19 infection; these subjects were stratified according to the number of days reported with symptoms in the acute phase (AP), between 1 and 14 days, or hyperinflammatory phase (HP) more than 14 days (Datta et al., 2020). To ensure the quality and reliability of the data, we conducted several preliminary analyses before the formal analyses. First, we use descriptive statistics to assess the frequencies, percentages, central tendency, and dispersion measures. Parametric and non-parametric contrast tests (χ^2^, Kruskal Wallis *H* test) were used depending on the normality (checked using Kolmogorov - Smirnov test) and homogeneity of variances (Levene test).

Furthermore, the duration elapsed between infection (COVID-19 diagnosis) and the cognitive evaluation, hours of reading, and exercise time were evaluated using a one-way ANOVA, but no significant difference was found. We run this considering that lifestyle factors may impact cognition in some cohorts (Chino et al., 2022). The effect of age and education was assessed with a linear regression analysis. The performances were significantly different according to educational level and age. Considering this effect, the second step evaluated the differences between cognitive performance using an ANCOVA analysis with age and education as covariates in all comparisons, adjusting the results for multiple comparisons (Bonferroni correction). Finally, the sample was assessed with an ANCOVA analysis, dividing the model according to sex and adjusting for the covariables (age and education) in males (*n* = 61) and women (*n* = 92). Statistical analysis was performed with SPSS version 24 (SPSS, Inc., Armonk, NY, USA) and GraphPad version 8. Significant results are reported with *p <* 0.05* and *p <* 0.01*.

## 3 Results

Among the sample, the control sample’s mean age was 40.62 years old (±18.34), and 40.5% of the participants were females. The largest group (45.2%) had more than 15 years of education; no schooling represented 2.4% of the sample. The control mean general cognitive performance score was 27.67 (±1.79). The participants with previous COVID infection were divided according to the number of days with symptoms of two groups: acute infection participants (AP) vs. post-acute hyperinflammatory participants (HP) (Matschke et al., 2020). There were no differences in sex (χ*^2^ =* 0.209, *p =* 0.901), age (*F:*1.344, *p =* 0.264), or education (*F:*0.035, *p =* 0.965) between these three groups (control, AP and HP) (see Supplementary Table S1). Sociodemographic characteristics for the women’s and men’s groups are presented in Table 1. The final sample included 154 participants (60% female). The results of the descriptive analysis of the participant’s performance are displayed in Table 1. All participants underwent evaluation within a time frame ranging from 3 to 30 months after their COVID-19 infection, during which symptoms were documented. This indicates that the participants did not report any precise or pertinent symptoms during the evaluation. Nevertheless, the previously reported COVID-19 symptoms, in conjunction with PCR/Antigen test results, were used to categorize the participants into distinct study groups.

**Table 1:** Sociodemographic characteristics of the sample by sex.

	All			*p-value*	Men			*p-value*	Women			*p-value*
	**Control**	**AP**	**HP**		**Control**	**AP**	**HP**		**Control**	**AP**	**HP**	
*N*	**42**	**74**	**38**		**17**	**28**	**16**		**25**	**46**	**22**	
Age	40.62 ± 18.34	35.62 ± 14.41	37.11 ± 15.44	0.798	37.29 ± 16.23	34.64 ± 14.51	33.19 ± 16.89	0.981	42.88 ± 19.63	36.22 ± 14.48	39.95 ± 10.01	0.51
Years of education (years)	13.36 ± 3.57	13.49 ± 2.63	13.5 ± 2.09	0.0428	14.59 ± 1.81	12.64 ± 3.31	12.81 ± 2.07	0.73	12.52 ± 4.21	14.00 ± 1.98	14.00 ± 2.00	0.324
Reading time (hours)^a^	3.67 ± 5.90	6.65 ± 10.71	3.08 ± 4.73	0.481	2.38 ± 4.31	9.43 ± 13.13	3.31 ± 5.38	0.464	4.54 ± 6.71	4.96 ± 8.65	2.91 ± 4.32	0.717
Physical activity (hours) ^a^	1.54 ± 2.00	1.71 ± 4.71	0.76 ± 1.42	0.601	1.59 ± 1.33	2.38 ± 6.81	0.97 ± 1.81	0.79	1.50 ± 2.38	1.30 ± 2.77	0.61 ± 1.07	0.374
Time between infection and cognitive evaluation.	473.28 ± 275.86	481.57 ± 245.55	484.59 ± 259.33	0.985	431.25 ± 302.54	510.48 ± 219.10	469.28 ± 294.67	0.69	502.94 ± 260.75	464 ± 261.54	497.00 ± 237.28	0.841
General Cognitive Performance	27.67 ± 1.79	27.74 ± 2.03	26.58 ± 2.37	0.066	27.82 ± 1.70	28.07 ± 2.00	26.31 ± 2.55	0.116	27.56 ± 1.87	27.54 ± 2.04	26.77 ± 2.27	0.374
Working memory (EF)	9.95 ± 2.65	9.72 ± 2.83	8.18 ± 2.24	**0.013**	11.12 ± 2.09	10.18 ± 2.89	7.50 ± 1.80	**0.001**	9.16 ± 2.73	9.43 ± 2.79	8.68 ± 2.44	0.574
Planning (EF)	53.45 ± 22.0	68.19 ± 25.52	99.66 ± 36.94	**<0.001**	54.35 ± 13.57	65.71 ± 25.60	113.69 ± 37.64	**<0.001**	52.84 ± 26.51	69.70 ± 25.64	89.45 ± 33.67	0.100
Cognitive Flexibility (EF)	90.83 ± 31.18	141.41 ± 125.14	150.71 ± 149.39	0.096	89.35 ± 25.27	117.43 ± 55.54	181.94 ± 227.07	**0.013**	91.84 ± 35.11	156.00 ± 151.59	128.00 ± 34.75	0.188

Table provides the mean values as mean ± standard deviation (SD) for the characteristics of the participants as well as the variables used for correlation analyses. These include age (in years), years of education, reading time (hours), physical activity (hours), time between infection and cognitive evaluation (in days), and cognitive performance in different variables. The ANOVA test was used to compare within-group differences. *F*-statistic, *F* (2,151). Statistically significant difference at *p* < 0.05.

**Figure 1:**
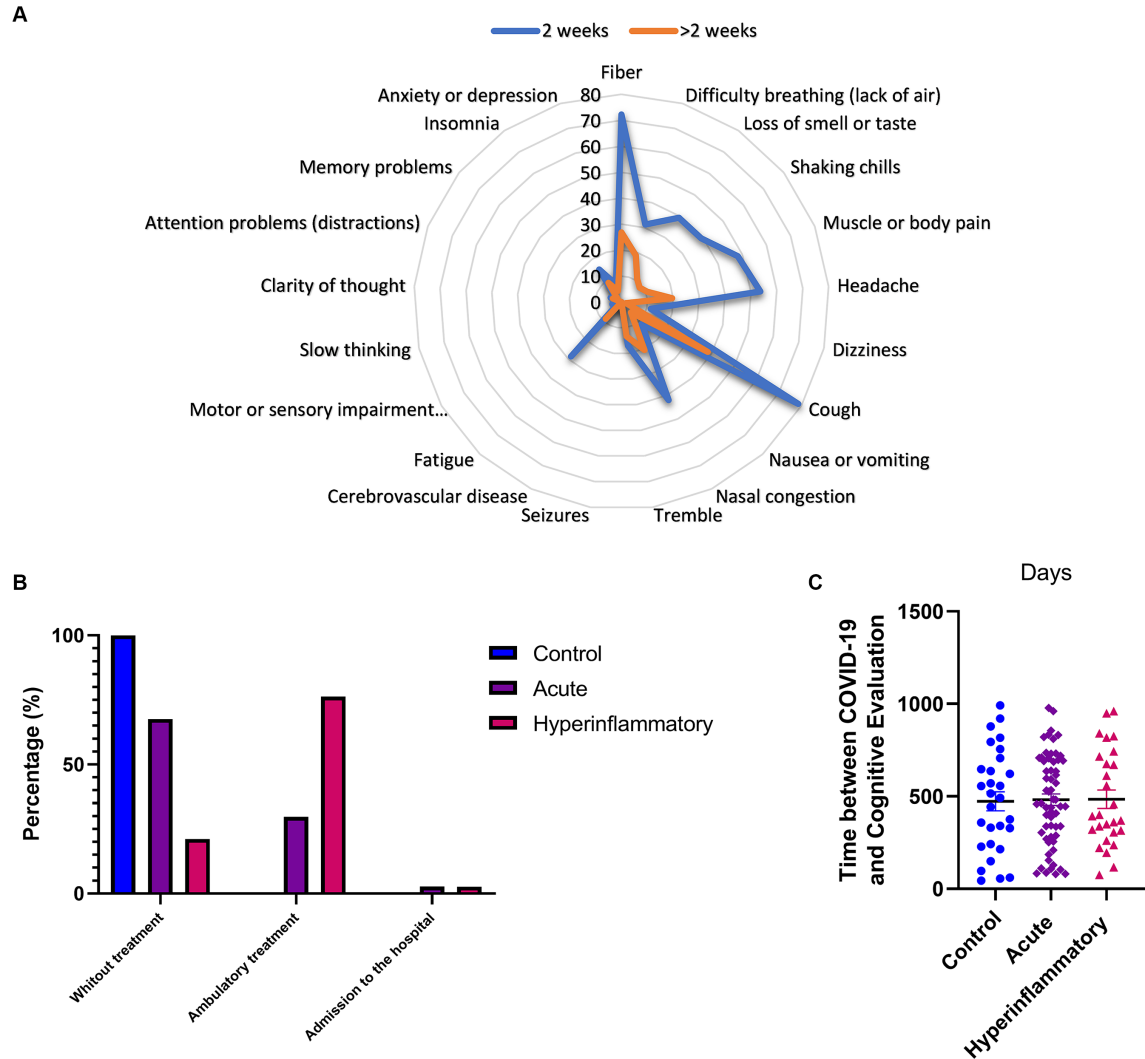
COVID-19 characteristics related to symptoms, treatment, and time between diagnosis and cognitive evaluation. **(A)** More frequent symptoms divided by presentation time. **(B)** Percentage of participants and the treatments they received. **(C)** Elapsed duration between infection and the cognitive evaluation in days. Significant results are reported with *p <* 0.05*, *p <* 0.01**, and *p <* 0.001***.

Regarding the symptoms displayed by the participants, Figure 1A shows a radial graph with the more frequent divided by presentation time (less than 2 weeks and more than 2 weeks). The most frequent was cough, 78.6%, followed by fiber (72.3%). Headache was presented in 53.6%, fatigue (28.6%), and insomnia (15.2%). Cognitive-related, slow thinking was indicated in 3.6% of the participants; attention problems and distraction were 4.5%. For over 2 weeks, the cough displayed 38.4%, fiber (26.8%), and headache (19.6%). Other cognitive symptoms were reduced as fatigue (8.9%), slow thinking (0.9%), and attention problems (3.6%).

Figure 1B shows the percentage of participants and the treatments they received. For example, 29.7% of the participants in the acute phase had ambulatory treatment, while 2.7% needed admission to the hospital. In contrast, 76.3% of those in the hyperinflammatory phase needed ambulatory treatment, and 2.6% were admitted to a hospital. Control participants do not need ambulatory treatment. Figure 1C displays the elapsed duration between infection (from COVID-19 diagnosis) and the cognitive evaluation in days. The mean time in the controls was (473.3 ± 275. 9 days), AP group (481.6 ± 245.6 days), and HP group (484.6 ± 259.3 days). There was no difference in the groups about this time (*F:*0.837, *p =* 0.435).

**Figure 2:**
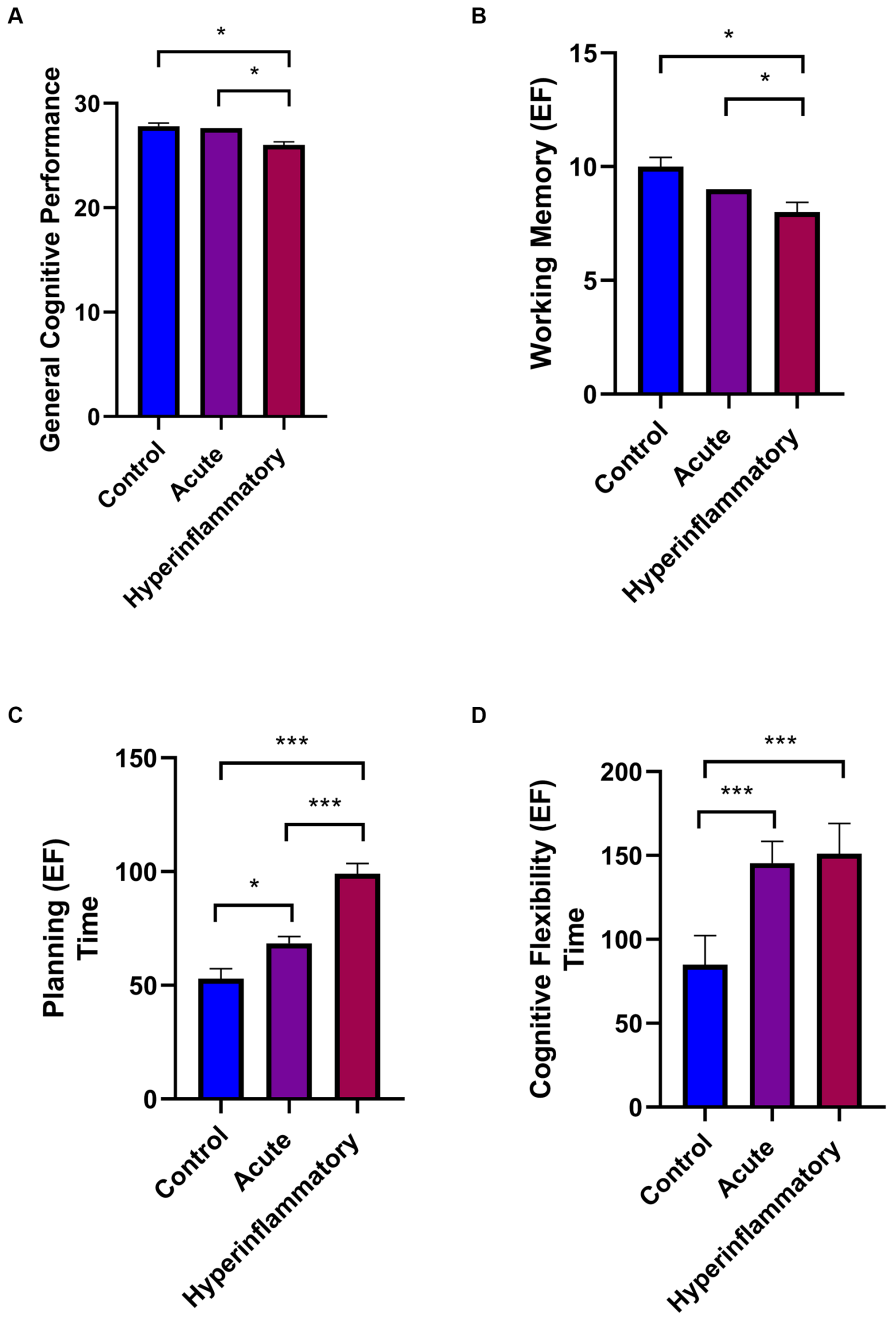
Cognitive general performance and executive functioning of all participants between COVID-19 phases. **(A)** General Cognitive performance. **(B)** Working Memory. **(C)** Planning time. **(D)** Cognitive Flexibility Time. Significant results are reported with *p <* 0.05*, *p <* 0.01**, and *p <* 0.001***.

The first approach in all the samples showed significant differences between the controls and AP in executive function, controlled by age and education (see descriptive data in Supplementary Table S1, S2). We found differences in planning; *p =* 0.02, *ηp^2^:* 0.279; and flexibility; *p =* 0.02, *ηp^2^*: 0.06 (see Figure 2). When comparing the control and HP groups, significant differences appear in general cognitive performance (*p =* 0.02, *ηp^2^:* 0.062), working memory (*p =* 0.01, *ηp^2^:* 0.068), and executive function (Planning; *p <* 0.001, *ηp^2^:* 0.279; Flexibility; *p =* 0.03, *ηp^2^:* 0.060), showing better scores in control subjects. In both cases, increased timing indicates worse performance.

**Figure 3:**
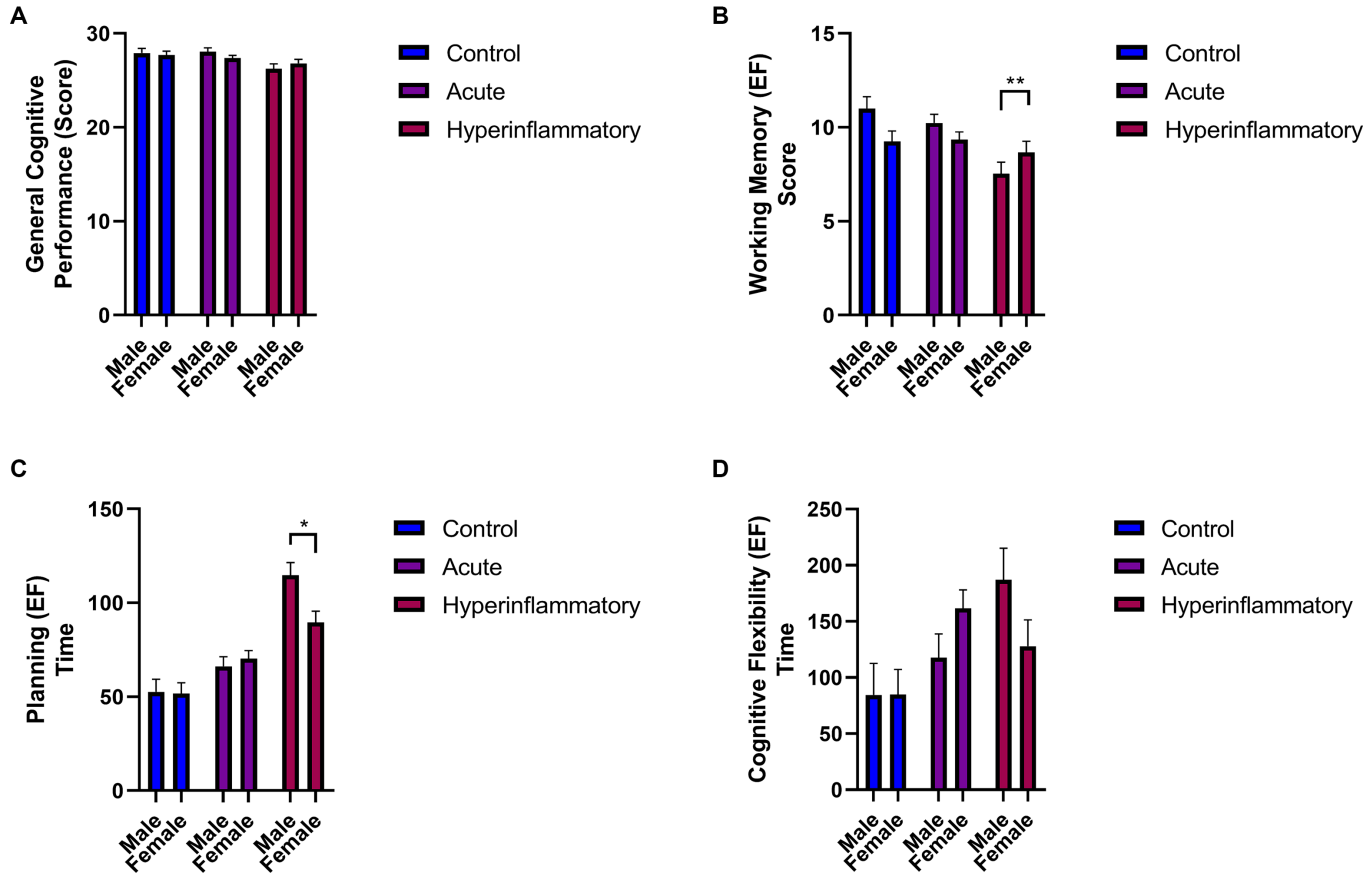
Comparisons of cognitive performance and executive functioning by sex. **(A)** General cognitive Performance. **(B)** Working Memory Score. **(C)** Planning time. **(D)** Cognitive Flexibility Time. Significant results are reported with *p <* 0.05*, *p <* 0.01**, and *p <* 0.001***.

There were also significant differences between patient groups (AP vs. HP) in general cognitive performance (*p =* 0.02, *ηp^2^:* 0.062), working memory (*p =* 0.02, *ηp^2^:*0.068), and planning (*p <* 0.001 *ηp^2^:* 0.279), with the scores higher in the subjects with less than 14 days of illness. Regarding sex differences (see Figure 3), they only appear in the HP group in working memory (*p =* 0.003; *ηp^2^:* 0.227) and planning (executive function; *p =* 0.01; *ηp^2^:* 0.172). HP males showed significantly worse performance in these cognitive domains compared to females.

**Figure 4:**
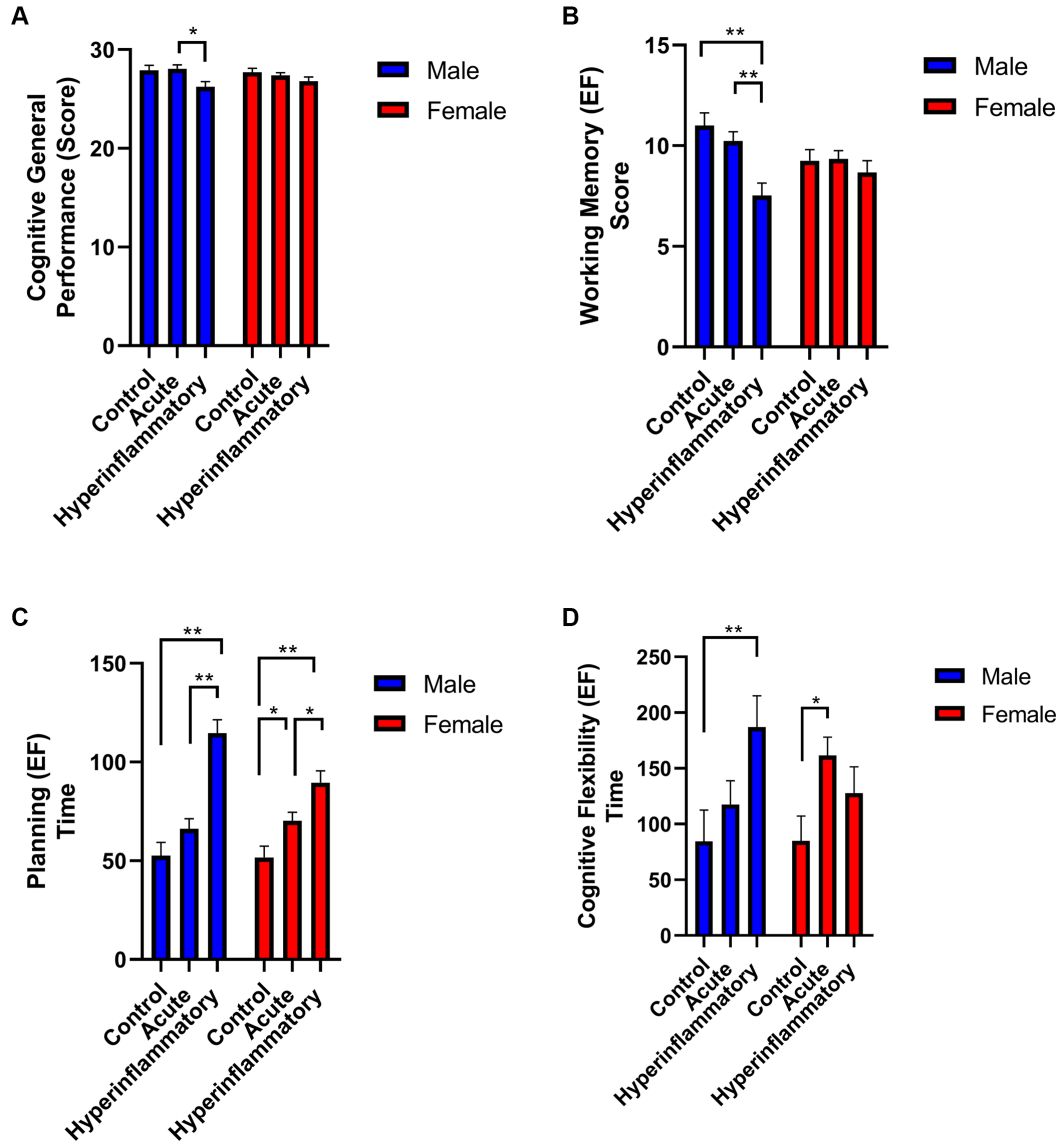
Cognitive general performance and executive functioning comparisons between COVID-19 phases by sex. **(A)** General cognitive Performance. **(B)** Working. Significant results are reported with *p <* 0.05*, *p <* 0.01**, and *p <* 0.001***.

We found specific differences when we compared male and female performance regarding the COVID-19 phase (see Figure 4). For example, in the general cognitive performance of males, the differences appear only in the AP vs. HP group (*p =* 0.02; *ηp^2^:* 0.136); working memory showed significant differences in controls vs. HP males (*p =* 0.001; *ηp^2^:* 0.245) and between patients (*p =* 0.002; *ηp^2^:* 0.245). Regarding executive function, these outcomes persisted between controls vs. HP (Planning; *p <* 0.001, *ηp^2^:* 0.464; Flexibility; *p =* 0.04, *ηp^2^:* 0.11) and between AP vs. HP males, only in the planning subdomain (*p <* 0.001, *ηp^2^:* 0.464).

In the women sample, the significant differences only appear across the AP and HP groups in the executive function domain like planning (Controls vs. AP: *p =* 0.03, *ηp^2^:* 0.192) and cognitive flexibility when compared control vs. AP group (*p =* 0.02, *ηp^2^:* 0.08). When we compared controls to the HP group, we only found differences in planning (Controls vs. HP: *p <* 0.001, *ηp^2^:* 0.192). Between COVID-19 phases in females (AP vs. HP), we identify differences in planning: *p =* 0.03, *ηp^2^:* 0.08.

## 4 Discussion

To our knowledge, this is the first cross-sectional study in Peru about cognitive health in a cohort of participants infected with SARS-COV-2 with symptoms in the acute phase and hyperinflammatory phase. Our study took place in Chiclayo, Peru, a country with one of the highest COVID-19 mortality rates in the world and one of the most severe COVID-19 lockdown restrictions worldwide (Vázquez-Rowe and Gandolfi, 2020).

The neuropsychological performance profile obtained in our study shows significant differences for the task evaluated in our battery. These tests measured general cognitive performance, working memory, and executive function, with planning performance as the most impaired function across all the control and patient group comparisons. Those individuals who reported symptoms in the acute phase of COVID-19 showed inferior cognitive performance in executive function, working memory, and planning compared to the control group. Similarly, individuals who reported symptoms in the hyperinflammatory phase of COVID-19 also displayed significantly poorer cognitive performance in overall cognitive function, working memory, and executive function compared to the control group. These results align with previous and simultaneous studies carried out after the pandemic. For example, a 2-year retrospective study with more than 1,487,712 participants worldwide of different ages found that COVID-19 increases the risk of neurologic and psychiatric sequelae in the following weeks and months after infection (Taquet et al., 2022). Even though most of these participants did not show severe symptoms from the COVID-19 infection, and only a small proportion needed hospitalization or ICU admission, the detection of the virus in their health records via PCR or antigen tests indicates to us that the COVID-19 infection can have an impact on neurocognition even with mild or moderate symptoms.

Moreover, aligned with our study, a systematic review highlighted scarce evidence assessing the consequences of COVID-19 on cognition. However, the results appear to suggest some form of cognitive deficits associated with COVID-19 in the acute and short-term follow-up phase, revealing that people with COVID-19 had poorer general cognitive functioning (measured with RUDAS-P) compared to people without COVID-19 between assessment in the acute phase and 6 months after infection (Crivelli et al., 2022). In this sense, objective measures show that executive functions (such as inhibition, updating, and set-shifting) also get impaired, and effects range from small to large, and generally, decreases in performance are related to advanced age and illness severity (Velichkovsky et al., 2023). When the studies explore racial differences in reports of long COVID and characterize the magnitude and differences in long COVID cognitive symptomatology, Black participants and Hispanic participants demonstrated a higher likelihood of developing long COVID than their White participants counterparts (Jacobs et al., 2023). Racial differences in the development of long COVID may be partially explained by racial differences in the likelihood of having private health insurance, which is believed to be a protective factor from developing long COVID; they had more probability of being infected, more likely to be hospitalized and less likely to have access to testing (Berger et al., 2021). An analogous situation was expected in Latin America (Ibanez et al., 2020).

Furthermore, our study exposed cognitive performance disparities influenced by gender. Within the hyperinflammatory phase, men experienced significantly compromised working memory and planning abilities compared to their counterparts in the control group. Besides, our results align with other studies showing increased severity after COVID-19 infection in males (Pradhan and Olsson, 2020; Henneghan et al., 2022). On the other hand, women in both the acute and hyperinflammatory phases exhibited significantly weakened executive function, specifically in planning and cognitive flexibility, when contrasted with women in the control group. These results align with previous reports. For example, a nationally representative sample also found females more likely to develop long COVID than their male counterparts (Jacobs et al., 2023). This report is like previous findings that have identified being female as a risk factor for developing long-term COVID controlling for the severity of the disease (Bai et al., 2022), probably explained by immune response differences. While males tend to be more susceptible to viral infections than females, due to females’ higher response to viral infections, they tend to have worse disease outcomes (Kopel et al., 2020). Additionally, the disproportionate impact of lifestyle changes brought upon by the COVID-19 pandemic compared to males, particularly in Peru (Antiporta et al., 2021), in which females appear to have worse post-COVID outcomes associated with life stressors that may translate into more significant complications among those with long COVID symptomatology (Crivelli et al., 2022).

In terms of limitations, the study’s sample size is noteworthy, which could affect the applicability of findings to broader populations. While supplying insights within a specific period, the study’s retrospective design hinders the exploration of temporal trends and causal relationships. Furthermore, the absence of extended following of participants over time limits our ability to fully grasp the lasting consequences of the virus on neurocognitive functioning. On the other hand, a small number of patients were hospitalized, either because they required O2 therapy or were admitted to the ICU (see Figure 1B). Among these, a very small portion of our sample was classified into the Acute or HP group. No significant differences were found, and no outliers were identified in subsequent comparisons. Nonetheless, we considered that hospitalization may have influenced cognition through mechanisms such as hypoxia, but further analysis and an increased sample size are needed.

Collectively, the findings of this study propose that COVID-19 might exert an adverse influence on cognitive performance, primarily focusing on executive function (planning), even in individuals with mild or moderate symptoms, and that this alteration could be detected up to 30 months after the first infection. Moreover, the research suggests that the cognitive repercussions of COVID-19 could be more pronounced in men and those undergoing the illness’s acute or hyperinflammatory phases. Considering these observations, the implementation of neuropsychological evaluations assumes significance, both for diagnostic purposes and for quantifying their severity and long-term prognostic implications. The intricate and personalized assessment of cognitive impairment holds the potential to inform the formulation of tailored treatment strategies.

## Data availability statement

The raw data supporting the conclusions of this article will be made available by the authors, without undue reservation.

## Ethics statement

The Universidad Señor de Sipán S.A.C Research Ethics Committee reviewed and approved the study involving humans with the Code N.° 0086-27092022-CIEI. The study was conducted in accordance with the local legislation and institutional requirements. The participants provided their written informed consent to participate in this study.

## Author contributions

JZ-V: Conceptualization, Formal analysis, Funding acquisition, Investigation, Methodology, Project administration, Resources, Software, Supervision, Validation, Visualization, Writing – original draft, Writing – review & editing. HA-N: Data curation, Investigation, Project administration, Writing – original draft, Writing – review & editing. LP-F: Investigation, Writing – original draft, Writing – review & editing. RA-M: Investigation, Writing – original draft, Writing – review & editing. MC: Investigation, Writing – original draft, Writing – review & editing. DB-D: Investigation, Writing – original draft, Writing – review & editing. MM-S: Investigation, Writing – original draft, Writing – review & editing. VG-M: Investigation, Writing – original draft, Writing – review & editing. EÁ-B: Investigation, Writing – original draft, Writing – review & editing. TA-C: Investigation, Writing – original draft, Writing – review & editing. EA-S: Investigation, Writing – original draft, Writing – review & editing. MO-P: Investigation, Writing – original draft, Writing – review & editing. MC-O: Project administration, Writing – original draft, Writing – review & editing. PC: Investigation, Writing – original draft, Writing – review & editing. BC-V: Data curation, Formal analysis, Software, Visualization, Writing – original draft, Writing – review & editing. CP-M: Investigation, Writing – original draft, Writing – review & editing. CC: Investigation, Writing – original draft, Writing – review & editing. NC: Methodology, Supervision, Validation, Visualization, Writing – original draft, Writing – review & editing. AI: Supervision, Validation, Visualization, Writing – original draft, Writing – review & editing.
