# Supplementary material for: The impact of COVID-19 post-infection on the cognition of adults from Peru
Source: Front Psychol. 2024 Jun 25;15:1325237. doi: 10.3389/fpsyg.2024.1325237 (PMC11232419; doi:10.3389/fpsyg.2024.1325237)
Supplement: Supplementary file 1 [file Data_Sheet_1.docx]

**Supplementary Table 1.**

**Cognitive general performance and executive functioning comparisons between participants**

|  | | **All** | | | **Men** | | | **Women** | | |
| --- | --- | --- | --- | --- | --- | --- | --- | --- | --- | --- |
|  |  | **Control** | **AP** | **HP** | **Control** | **AP** | **HP** | **Control** | **AP** | **HP** |
|  | ***N*** | **42** | **73** | **38** | **17** | **28** | **16** | **25** | **45** | **22** |
| **General Cognitive Performance** | **M** | 27.67 | 27.71 | 26.58 | 27.82 | 28.07 | 26.31 | 27.56 | 27.49 | 26.77 |
|  | **(SD)** | 1.79 | 2.02 | 2.37 | 1.7 | 1.99 | 2.55 | 1.87 | 2.03 | 2.27 |
|  | **Madj** | 27.8 | 27.64 | 26.56 | 27.91 | 28.06 | 26.24 | 27.72 | 27.38 | 26.82 |
|  | **(SE)** | 0.3 | 0.23 | 0.32 | 0.51 | 0.39 | 0.51 | 0.39 | 0.29 | 0.41 |
| **Working memory**  **(EF)** | **M** | 9.95 | 9.7 | 8.18 | 11.12 | 10.18 | 7.5 | 9.16 | 9.40 | 8.68 |
|  | **(SD)** | 2.65 | 2.85 | 2.24 | 2.09 | 2.89 | 1.79 | 2.73 | 2.81 | 2.44 |
|  | **Madj** | 10 | 9.67 | 8.19 | 11.007 | 10.23 | 7.53 | 9.26 | 9.35 | 8.67 |
|  | **(SE)** | 0.41 | 0.31 | 0.43 | 0.62 | 0.46 | 0.61 | 0.55 | 0.41 | 0.58 |
| **Planning (EF)** | **M** | 53.45 | 68.19 | 99.66 | 54.35 | 65.71 | 113.69 | 52.84 | 69.73 | 89.45 |
|  | **(SD)** | 22.00 | 25.7 | 36.94 | 13.57 | 25.6 | 37.64 | 26.51 | 25.93 | 33.67 |
|  | **Madj** | 52.93 | 68.48 | 99.68 | 52.53 | 66.22 | 114.74 | 51.65 | 70.34 | 89.57 |
|  | **(SE)** | 4.35 | 3.29 | 4.55 | 6.75 | 5.09 | 6.73 | 5.74 | 4.24 | 6.03 |
| **Cognitive Flexibility (EF)** | **M** | 90.83 | 142.23 | 150.71 | 89.35 | 117.43 | 181.94 | 91.24 | 91.64 | 90.59 |
|  | **(SD)** | 31.18 | 125.81 | 149.39 | 25.27 | 55.53 | 227.07 | 6.67 | 5.07 | 6.05 |
|  | **Madj** | 84.87 | 145.44 | 151.14 | 84.49 | 117.51 | 186.96 | 84.81 | 161.64 | 127.87 |
|  | **(SE)** | 17.35 | 13.12 | 18.14 | 28.13 | 21.24 | 28.06 | 22.28 | 16.46 | 23.38 |

***Note.*** Supple Table 1 provides the mean adjusted ± standard deviations and standard error for cognitive domains. The results are provided for the whole sample and stratified by sex (men or women).

**Supplementary Table 2.**

**Cognitive general performance and executive functioning comparisons using ANCOVA**

|  | All sample | | | Men | | | Women | | |
| --- | --- | --- | --- | --- | --- | --- | --- | --- | --- |
|  | **F-Statistic** | **p-value** | **Effect size** | **F-Statistic** | **p-value** | **Effect size** | **F-Statistic** | **p-value** | **Effect size** |
| General Cognitive Performance | 4,897 | 0,009 | 0,062 | 4,393 | 0,017 | 0,136 | 1,274 | 0,285 | 0,028 |
| Working memory | 5,437 | 0,005 | 0,068 | 9,103 | <0.001 | 0,245 | 0,470 | 0,627 | 0,011 |
| Planning (EF) | 28,651 | <0.001 | 0,279 | 24,213 | <0.001 | 0,464 | 10,335 | <0.001 | 0,192 |
| Cognitive Flexibility (EF) | 4,724 | 0,010 | 0,06 | 3,460 | 0,038 | 0,110 | 3,792 | 0,026 | 0,080 |

*Note.* Statistical results of the ANCOVA used the age and education as covariates. Effect size is reported as partial n2.
